# Resource-oriented music therapy for psychiatric patients with low therapy motivation: Protocol for a randomised controlled trial [NCT00137189]

**DOI:** 10.1186/1471-244X-5-39

**Published:** 2005-10-31

**Authors:** Christian Gold, Randi Rolvsjord, Leif Edvard Aaro, Trond Aarre, Lars Tjemsland, Brynjulf Stige

**Affiliations:** 1 Faculty of Health Studies, Sogn og Fjordane University College, 6823 Sandane, Norway; 2 Nordfjord Psychiatry Centre, 6770 Nordfjordeid, Norway; 3 University of Bergen, 5020 Bergen, Norway; 4 Stavanger University Hospital, 4068 Stavanger, Norway

## Abstract

**Background:**

Previous research has shown positive effects of music therapy for people with schizophrenia and other mental disorders. In clinical practice, music therapy is often offered to psychiatric patients with low therapy motivation, but little research exists about this population. The aim of this study is to examine whether resource-oriented music therapy helps psychiatric patients with low therapy motivation to improve negative symptoms and other health-related outcomes. An additional aim of the study is to examine the mechanisms of change through music therapy.

**Methods:**

144 adults with a non-organic mental disorder (ICD-10: F1 to F6) who have low therapy motivation and a willingness to work with music will be randomly assigned to an experimental or a control condition. All participants will receive standard care, and the experimental group will in addition be offered biweekly sessions of music therapy over a period of three months. Outcomes will be measured by a blind assessor before and 1, 3, and 9 months after randomisation.

**Discussion:**

The findings to be expected from this study will fill an important gap in the knowledge of treatment effects for a patient group that does not easily benefit from treatment. The study's close link to clinical practice, as well as its size and comprehensiveness, will make its results well generalisable to clinical practice.

## Background

Music therapy is defined as a systematic process where the therapist helps the client to promote health, using musical experiences and the relationships that develop through them [1]. It is often perceived as a psychotherapeutic method where musical interaction, in addition to verbal discussion, is used as a means of communication and expression. The aim of music therapy is to help people with mental health problems to develop relationships and to address issues they may not be able to by using words alone. Results from a Cochrane review showed that music therapy helps people with schizophrenia to improve their global state, mental state and social functioning in the short to medium term [2]. The review suggested that there is a need for studies examining the effects of music therapy over a longer term. Furthermore, studies are needed to examine the effectiveness of music therapy in clinical practice, and to further explore the psychological 'mechanisms' through which music therapy works.

Music therapy is usually not tailored to a specific diagnosis. Rather, contents of therapy are negotiated with the patient within the process of therapy, based on a variety of individual traits. It has been suggested that factors unrelated to psychiatric diagnosis, specifically therapy motivation, be considered when specifying, prescribing, and evaluating psychotherapy [3]. Psychotherapy may not work if patients are not motivated for it [4-6]. In music therapy, the use of music (i.e. playing or listening to music) itself can often be a motivating factor for patients who may otherwise not be motivated for psychotherapy [7]. Therefore, a low motivation for (other) therapy can become a reason for referral of a patient to music therapy, and such factors may at times be more important than the patient's primary diagnosis. However, there is a scarcity of research addressing the effects of music therapy for patients with low therapy motivation. We found only one randomised study on music therapy for depression where the authors described that the majority of the participants had previously failed to respond to verbal psychotherapy [8].

The problem of low motivation may sometimes be due to a lack of insight and will often lead to poor therapy outcome. It has been described for a variety of disorders, including schizophrenia [9-12], depression and bipolar disorder [11,13], and psychosomatic disorders [14,15]. Music therapy is often recommended for such patients and may have something unique to offer which is worth exploring. A randomised study is needed to examine the potential of music therapy for this under-researched but clinically important population.

Resource-oriented music therapy for people with mental health problems is oriented towards the client's resources, strengths and potentials, rather than primarily on problems and conflicts, and emphasizes collaboration and equal relationships [16,17]. Such a perspective to music therapy builds on a contextual understanding of therapeutic processes [6,18,19], the philosophy of empowerment [20,21], and positive psychology [22]. In music therapy, music may be seen as a central resource for the patient, but a resource-oriented approach will also emphasise the patient's resources in the verbal discussions taking place within the music therapy sessions [17]. Goals of resource-oriented music therapy with people with mental health problems include, among others, the ability to feel and express emotions, to build and sustain relationships to others, and to develop interest and motivation. Therefore the goals of the therapy are closely related to what has been described as negative symptoms in mental health research [2,23].

### Objectives

The objectives of this study are as follows:

1.) To determine whether resource-oriented music therapy helps psychiatric patients who have a low therapy motivation and a willingness to work with music to reduce their level of negative symptoms (primary study outcome).

2.) To determine whether the therapy helps the patients to improve in the following secondary outcomes:

(a) secondary outcomes of general relevance for the patient: general symptoms; general functioning; clinical global impressions.

(b) secondary outcomes specifically linked to the assumed mechanisms of the therapy: interest in music; motivation for change; self-efficacy; self-esteem; vitality; affect regulation; relational competence; actual social relationships.

3.) Provided that significant effects are found: To determine whether general outcomes are mediated by specific outcomes.

## Methods

### Participants

The study will include adult patients with mental disorders who have a low motivation for therapy, as specified below. Criteria for in- and exclusion will be assessed by the ward psychiatrist, based on information collected by the clinical team on the ward.

#### Inclusion criteria

##### (a) Diagnosis F1 to F6

Participants must have a non-organic mental disorder (F1 to F6 according to ICD-10), as assessed by a psychiatrist at a participating centre. The inclusion of such a broad range of mental disorders is based on the finding that mechanisms of psychotherapy are not specifically linked to diagnosis [6]. This broad range of diagnoses will also improve external validity, which is often not optimal in randomised trials which have too narrow inclusion criteria [24].

##### (b) Low therapy motivation

This is the main inclusion criterion for the study. Patients are often referred to music therapy because they have a low therapy motivation and music can be motivating for them. The specific reasons for this low therapy motivation may vary. Some patients may have insufficient insight into having a mental health problem. Others may have insight about having a problem but fail to acknowledge psychosocial components. These patients may demand a 'medication cure' and state that they do not believe in talking. Other patients may state that they do not feel comfortable with talking about emotions and personal problems. Patients may also have low therapy motivation because they did not improve from therapy previously.

##### (c) Willingness to work with music

Participants will be included if they show a willingness to work with music in music therapy. They do not need to have an established interest in music, such as having learnt an instrument or enjoying listening to music, although this may be the case for some of them.

#### Exclusion criteria

##### (a) Severe mental retardation

The outcome measures include some self-reports and therefore participants who are unable to complete these cannot be included. Participants need to be cognitively able to complete a self-report questionnaire.

##### (b) Severe life-threatening somatic illness

Participants with a severe life-threatening somatic illness will not be included because the dynamics of such illness would have such a strong influence on the course of therapy that it would be highly questionable to pool them with other patients.

### Interventions

Participants will be randomly assigned to two groups (details in next section). The interventions for both groups will be provided and monitored over the course of three months from randomisation.

#### Experimental group

##### (a) Music therapy

Participants assigned to the experimental group will receive individual sessions of resource-oriented music therapy. Two sessions per week will be offered, lasting each 45 minutes. Over the course of three months this corresponds to a maximum of 26 sessions. Previous research suggests that at least about 20 sessions are needed for music therapy to have an effect [2]. In cases where it is not possible to provide the maximum number of sessions, therapists should try to ensure that at least 18 sessions will be given within the three-month period. This may be the case when outpatients live too far from the centre to attend two times per week throughout the study period.

Music therapy will be provided in accordance with the principles of resource-oriented music therapy [16,17]. These principles describe general therapeutic attitudes and behaviours (e.g. focusing on the client's strengths and potentials) as well as specific attitudes within the musical interaction (e.g. tuning into the client's musical expression). Attitudes that should be avoided are also described, as well as attitudes that are acceptable but not necessary. Adherence to these principles and competence in their application [16,17,25] will be monitored in two ways. Therapists will rate their own behaviour at the end of every session. This is an efficient way of monitoring the complete course of therapy. To control for a possible subjective bias in these self-reports, randomly selected sessions will be videotaped and the therapist's adherence and competence will be assessed by independent raters. Half of all participants in the experimental group will be randomly selected for videotaping of one therapy session which will also be selected randomly.

##### (b) Standard care

Patients will continue to receive treatment as usual while receiving music therapy. What kind and what dose or frequency of other treatment they receive will be monitored by the ward clinician before randomisation and after 1, 3, and 9 months.

#### Control group

##### (a) Standard care

Patients will receive treatment as usual during the three-month study period. What kind and what dose or frequency of treatment they receive will be monitored by the ward clinician before randomisation and after 1, 3, and 9 months.

##### (b) After the study period: Optional music therapy

For ethical reasons and in order to keep participants in the control group motivated, they will be offered music therapy after the three-month study period. Setting (i.e. individual or group), frequency and duration need not be equivalent to the experimental therapy, but will be set according to clinical needs and possibilities. Adherence and competence will not be monitored.

### Study design

The study will use a single-blind (assessor blinded) randomised design with two parallel groups of equal size. Outcomes will be assessed at pretest (directly after inclusion, before randomisation), at an early intermediate time point (1 month after randomisation), posttest (3 months after randomisation), and six-month follow-up (9 months after randomisation).

The required sample size was calculated for the primary outcome, negative symptoms. We assumed an effect size slightly smaller than medium (f = 0.20, equivalent to d = 0.40), based on the results of our Cochrane Review [2]. For a one-way ANCOVA with one covariate (pretest values), α = 0.05, 80% power, and 36% variance explained by the covariate, the required sample size available for analysis (total number of valid cases) needs to be N = 2 * 65 = 130. In order to allow for 10% drop-outs, the total sample size will need to be N = 144. The actual power of the study may then be greater than 80% because of the additional intermediate assessment points [26].

**Figure 1:**
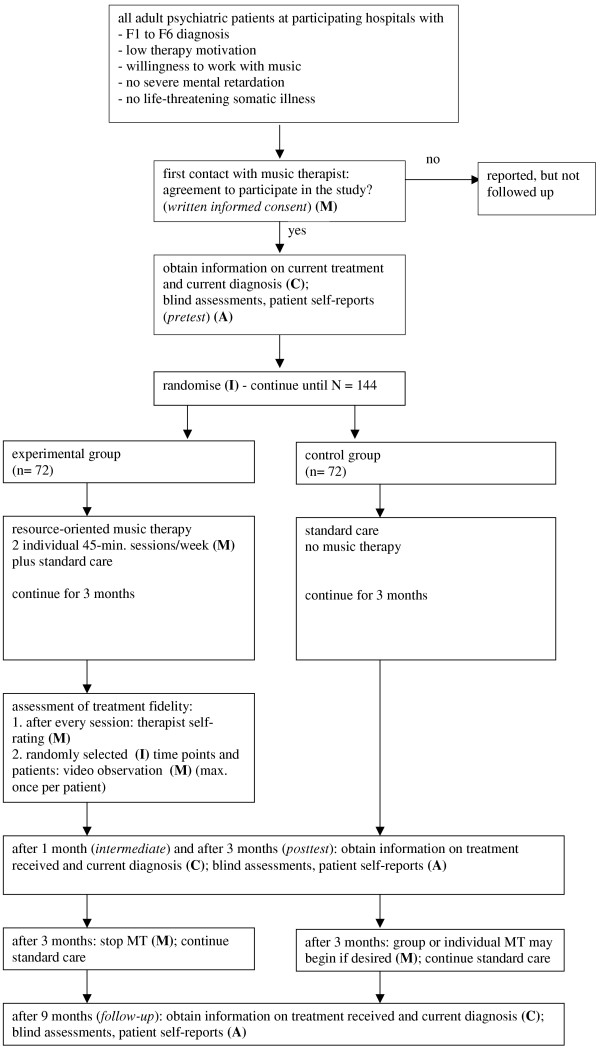
**Flow chart of the study design**. *Abbreviations: ***C **– ward clinician; **M **– music therapist; **I **– principal investigator; **A **– blind assessor; MT – music therapy.

After inclusion in the study and pretest assessment, the participants will be allocated to conditions using a computerised randomisation procedure, stratified by treatment centre and type of disorder (psychotic versus non-psychotic). This will be done by the principal investigator who has no direct contact to the patients in order to conceal the allocation from the involved clinicians. An overview of the study design is shown in Figure 1.

The following professionals will be involved in conducting the study and collecting data:

1. Ward clinician **(C)**: the clinician who has the primary responsibility for the patient at the hospital unit.

2. Music therapists **(M)**: academically qualified music therapists with clinical experience in music therapy in psychiatry and specifically trained in the use of the treatment principles for resource-oriented music therapy.

3. Principal investigator **(I)**: The first author (CG).

4. Blind assessor **(A)**: an experienced clinician who is not involved in the daily work at the patient's ward/hospital unit and therefore not aware of the patient's assigned treatment condition. The assessor will have received training in the use of the assessment instruments and will conduct a one-hour patient interview for each assessment. The success of blinding is verified with a separate question in the blind assessor questionnaire.

5. A local co-ordinator will help with the administrative side of the data collection. This person will supervise and facilitate the data collection process and ensure the reliable and timely transferral of information between hospital staff and principal investigator.

6. Other music therapists will assess treatment fidelity (adherence and competence) on the basis of the video recordings.

### Outcomes

The study will use blind ratings as well as self-reports. Standardised instruments with demonstrated validity, reliability and sensitivity to change will be applied whenever possible.

#### Primary outcome: Negative symptoms

The concept of negative symptoms has originally been developed mainly in relation to psychotic disorders but is considered relevant for other mental disorders as well [27,28]. Including affective flattening and blunting, poor social interaction and lack of interest, among others, it is reasonable to assume that processes within music therapy are directly linked to negative symptoms [2]. This outcome will be evaluated by a trained blind assessor using the Scale for the Assessment of Negative Symptoms (SANS) composite score [23]. Validity of the SANS scale has been demonstrated for a variety of mental disorders [27,28]. Interrater reliability, test-retest reliability, internal consistency and sensitivity to change following music therapy have been demonstrated for schizophrenic patients. In order to achieve reliable ratings, the assessor must be trained in the use of this scale.

#### Secondary outcomes of general relevance for the patient

• *General symptom level *will be assessed using the BSI-18 self-report scale with 18 items addressing anxiety, depression, and somatic complaints [29]. It has demonstrated concurrent and predictive validity as well as internal consistency in clinical and community samples.

• *General functioning *will be measured using a blind rating with the GAF [30]. The GAF is a widely used single-item scale which has demonstrated good predictive validity and interrater reliability.

• *Global clinical impressions *will also be evaluated by a blind assessor using the CGI scale [31]. It consists of two items and has been widely used to assess treatment outcomes in mental health because of its simplicity and intuitiveness.

#### Secondary outcomes specifically linked to the assumed mechanisms of music therapy

• *Interest in music: *We were unable to find a published scale that was appropriate for this outcome. Therefore we developed a self-report scale to measure interest in music. The scale has 11 Likert-scaled items assessing preferences for various uses of music, actual behaviours, and emotional responses to music. The scale is face-valid; its reliability will be determined from the study sample.

• *Motivation for change *will be measured using a modified version of the two URICA subscales precontemplation and contemplation [32,33]. Predictive validity, reliability, and sensitivity for change of this scale have been shown for a variety of mental disorders [34,35]. The scale includes 19 items. It will be used as a straightforward continuous measure in order to avoid the conceptual problems that are associated with the stages of change model [36]. In addition to this self-report instrument, a blinded assessment of the patients' general motivation will be included in the SANS avolition/apathy scale [23].

• *Self-efficacy *will be assessed using the modified Norwegian version of the General Perceived Self-Efficacy Scale [37]. This is a self-report measure with 10 Likert-scaled items that has demonstrated test-retest reliability and internal consistency in both clinical and non-clinical samples.

• *Self-esteem *will be measured using the Rosenberg Self-Esteem Scale [38], a self-report measure with 10 items. The scale has been used in many studies. Discriminant validity, test-retest reliability and internal consistency have been shown for patients with mental disorders.

• *Vitality *will be assessed using the vitality subscale of the SF-36 scale [39]. This is a self-report scale with 4 items. It has demonstrated discriminant validity and sensitivity to change in schizophrenic patients, and internal consistency and test-retest reliability have also been confirmed.

• *Affect regulation *will be measured with a blind rating using the SANS subscale affecting flattening and blunting [23]. The seven-item scale has good internal consistency. Interrater reliability is moderate. Sensitivity to change following music therapy has been demonstrated in schizophrenic patients.

• *Relational competence *will be assessed using the IIP-32 [40]. This self-report scale contains 32 items describing a variety of interpersonal problems. It has demonstrated internal consistency in psychotherapy patients and test-retest reliability in a non-clinical sample.

• *Actual social relationships *will be measured using both a self-report and a blinded assessment. The Q-LES-Q social relationships subscale [41] will be used in self-reports. It has 11 face-valid items and has demonstrated sensitivity to change, test-retest reliability and internal consistency in major depression. In addition, the blind assessor will complete the SANS anhedonia/asociality subscale [23]. The 5-item scale has demonstrated satisfactory interrater reliability, internal consistency, and sensitivity to change following music therapy in schizophrenic patients.

In total, the self-report questionnaire will consist of 114 items. The blind assessor will check the completed questionnaire for completeness and help the patient if necessary. The blind assessor questionnaire will consist of 28 items to be rated on the basis of a 1-hour clinical interview.

### Statistical analyses

The effects of treatment (Objectives 1 and 2) will be analysed using analysis of covariance methods and effect sizes with confidence intervals. Subgroup analyses are planned for psychotic versus non-psychotic disorders. No stopping rules or interim analyses are planned for this study. The primary analysis will be intention-to-treat.

Mediational processes (Objective 3) will be examined using structural equation modelling [42,43], which will address mediation of simultaneous as well as of subsequent change. Factor analysis will be used to examine structures in the adherence and competence ratings.

### Ethical issues

The study has been approved by the Regional Committees for Medical Research Ethics Western Norway (REK Vest).

### Time scale

The overall time frame for this project is from January 2004 to December 2007. Data collection is scheduled from April 2005 to June 2007, and we expect to be able to report the study's main results by December 2007.

## Discussion

### Relevance of the expected findings

The findings that can be expected from this study will be highly relevant because of its size and comprehensiveness and because of its close link to clinical practice. This study will have a much greater sample size than all previous studies on music therapy in the field of psychiatry to date. This will enable a more precise estimation of the effect of music therapy. The study will also be more comprehensive than previous studies in terms of how the treatment is defined and treatment fidelity measured, and in terms of the inclusion of potential mediator variables. This comprehensiveness will allow an evaluation of the processes and mechanisms leading to therapeutic change at a greater level of detail than in previous studies in the field. The close link to clinical practice will be ensured through the application of the flexible therapy manual, but also through the choice of the study population. The inclusion criteria for this study define a population that is often being referred to music therapy, and one that is in need of special attention. The results of the study will therefore be well generalisable to and relevant for clinical practice.

### Limitations

The main limitations of this study will include the lack of an alternative or 'placebo' therapy, the partial reliance on self-reports, and the broadness of the sample. Due to the lack of a placebo therapy, it could be argued that we did not control for the effect of receiving attention from a caring person. However, there are several arguments why a placebo therapy would not be adequate for this study. Most importantly, it has been argued that the metaphor of a placebo is conceptually inadequate in psychotherapy research, considering the discussion on common factors in psychotherapy [6]. It is therefore more appropriate to examine the specific processes and mechanisms of therapy by other means, such as the assessment of treatment fidelity and the assessment of mediator variables that we included in the design.

It may also be criticised that self-reports may be biased because of expectancy or social desirability effects. However, for some mental health outcomes, such as self-esteem, there is no alternative to self-reports. The main outcome for this study will however be rated by a blind assessor. Blinding of participants is not possible in psychotherapy studies.

A final criticism might concern the broad inclusion criteria. The study sample will be broad with regard to diagnoses, but the main inclusion criterion will be unrelated to diagnosis and well linked to an important clinical question. As this will improve generalisability, this last limitation can also as be regarded as one of the strengths of this study.

## Competing interests

CG, RR and BS are clinically trained music therapists.

## Authors' contributions

CG developed the background and design of the study, did the power calculation, helped to develop the therapeutic principles, and drafted the manuscript. RR conceived of the study and helped to draft the manuscript. LA helped with the design of the study and the power calculation. TA and LT participated in the design and the coordination of the study. BS helped in the conception and design of the study and in drafting the manuscript.

## Pre-publication history

The pre-publication history for this paper can be accessed here:


